# Community structure of tropics emerging from spatio-temporal variations in the Intertropical Convergence Zone dynamics

**DOI:** 10.1038/s41598-024-73872-0

**Published:** 2024-10-18

**Authors:** Gaurav Chopra, Vishnu R. Unni, Praveenkumar Venkatesan, Sara M. Vallejo-Bernal, Norbert Marwan, Jürgen Kurths, R. I. Sujith

**Affiliations:** 1 Department of Aerospace Engineering, Indian Institute of Technology Madras, Chennai, Tamil Nadu 600036 India; 2 Centre for Excellence for studying Critical Transitions in Complex Systems, Indian Institute of Technology Madras, Chennai, Tamil Nadu 600036 India; 3 Department of Mechanical and Aerospace Engineering, Indian Institute of Technology Hyderabad, Hyderabad, Telangana 502285 India; 4 Potsdam Institute for Climate Impact Research (PIK), Member of the Leibniz Association, Potsdam, 14412 Germany; 5 Institute of Geoscience, University of Potsdam, Potsdam, 14476 Germany; 6 Institute of Physics, Humboldt Universität zu, Berlin, 10117 Germany

**Keywords:** Complex networks, Climate sciences, Atmospheric science

## Abstract

The Intertropical Convergence Zone (ITCZ) is a narrow tropical belt of deep convective clouds, intense precipitation, and monsoon circulations encircling the Earth. Complex interactions between the ITCZ and local geophysical dynamics result in high climate variability, making weather forecasting and prediction of extreme rainfall or drought events challenging. We unravel the complex spatio-temporal dynamics of the ITCZ and the resulting teleconnection patterns via a novel tropical climate classification achieved using complex network analysis and community detection. We reduce the high-dimensional complex ITCZ dynamics into a simple yet insightful community structure that classifies the tropics into seven regions representing distinct ITCZ dynamics. The two largest communities, encompassing landmasses over the Northern and Southern hemispheres, are associated with coherent seasonal ITCZ dynamics and have significant long-range connections. Temporal analysis of the community structure highlights that the tropical Pacific and Atlantic Oceans communities exhibit substantial variation on multidecadal scales. Further, these communities exhibit incoherent dynamics due to atmosphere-ocean interactions driven by equatorial and coastal oceanic upwelling.

## Introduction

The Intertropical Convergence Zone (ITCZ) is a low-pressure region girdling the Earth, where heat and moisture-laden winds from the Northern and Southern hemispheres converge. The convergence enhances the convection of heat and moisture-laden winds to higher altitudes, where cooling and condensation lead to the formation of deep clouds and intense precipitation^1,2^. In fact, the ITCZ is often referred to as the ascending branch of the Hadley circulation^1,3,4^. The ITCZ follows the solar heating cycle and migrates in the meridional direction towards the warming hemisphere. Several monsoon systems, such as the Asian summer^3,5^, American^6,7^, African and Australian monsoons are associated with the arrival of the ITCZ in those regions. Precipitation during the monsoon seasons over these regions is the main source of fresh water for irrigation, drinking, household, and industrial applications.

The characteristics of the ITCZ are sensitive to regional interactions, circulations, the underlying surface, and local topographical conditions^1^. As a result, critical parameters such as cloudiness and precipitation associated with the ITCZ exhibit strong variability. Given the enormous societal impact of these monsoon systems, it is crucial to understand the spatio-temporal dynamics of the ITCZ, which is also key to substantially improving the forecasting of extreme events such as excessive rainfall and drought.

In this work, we explore the spatio-temporal dynamics of the ITCZ and propose a novel classification of the climate system in the tropics from the perspective of complex networks theory, which is a powerful framework for studying complex systems^8,9^. In the context of the climate system, the nodes of the complex network represent different geographical locations on Earth. Links represent the statistical association and similarity between climatological variables at these locations. Long-range associations and interactions, typically greater than 2500 km, are popularly referred to as teleconnections in climate science^10,11^. Such networks where connections are based on statistical similarity are classified as functional networks^10,12–14^. Here, we estimate the connections using Pearson correlation. Even though the climate is not a physical network, network analysis is an efficient statistical tool that unravels patterns of characteristic spatio-temporal dynamics, interactions, and teleconnections in the climate system^13,15,16^.

Over the recent years, functional networks have emerged as a potent tool for understanding the climate dynamics occurring on multiple spatio-temporal scales, ranging from meso to planetary and from subdaily to multiannual scales.^4,7,11,12,14,15,17^. Although the links in a functional network are based on statistical similarity, its topology has been shown to encode the climate dynamics that govern the studied phenomena. For instance, complex networks based on cross-correlations of ground-level temperature records exhibit a clear correspondence with the atmospheric Rossby wave^18^, an important mechanism for distributing energy across the planet. Complex networks based on nonlinear statistical measures are used for unravelling patterns of atmospheric teleconnections responsible for extreme climate events such as excessive rainfall^7,11^, and heat waves^19^. Cascading effects of atmospheric dynamics responsible for widespread heavy precipitation events have been revealed using climate networks and synchronization theory^20^. Network science is also utilized to explore the impact of various modes of climate variability, such as El Niño-Southern Oscillation (ENSO) and tropical interseasonal oscillation (ISO). Several studies have used network science to understand the global impact of ENSO^21,22^ and to enhance its forecasting and prediction^23^. Recently, the application of network science led to deepening our understanding of the role of ISO in modulating the interconnection between the Indian summer monsoon and the East Asian summer monsoon^24^. Functional networks are also constructed among domains, which are large regions consisting of a group of nodes (geographical locations) with coherent climate dynamics^25,26^. Such functional networks are also utilized for dimensionality reduction of large climate datasets^26^.

The ability to incorporate information regarding teleconnections is unique for complex networks, making it more beneficial than the traditional climate classification methods that mainly rely on knowledge or observation-based thresholding and statistical clustering^27^. Climate classification is an important approach that aids in monitoring and analyzing spatio-temporal patterns in the climate system^27–29^. Climate classifications such as the Köppen-Geiger classification^28,29^, which is based on the monthly air temperature, precipitation, and vegetation, and its variations are frequently used for validating global circulation models (GCMs)^30,31^, studying climate variability across multiple temporal scales^32,33^, climate change research^27^, and understanding vegetation distribution across the globe^29^, among many others^27^.

In this study, we apply community detection on a functional complex network constructed using outgoing longwave radiation (*OLR*) data to perform a network-based tropical climate classification. *OLR* is a good proxy for the cloudiness in the ITCZ and faithfully captures its spatio-temporal dynamics^3,5,34,35^. We apply a modularity optimizing community detection method, the Louvain algorithm^36^, on the *OLR* network to classify the tropics according to the variability in the spatio-temporal dynamics of the ITCZ. Community detection partitions the nodes into communities such that nodes within a community are densely connected, while connections with nodes outside are sparse. In the context of the climate system, such communities are interpreted as groups of geographical locations that are associated with similar physical climate processes, phenomena, and major climate modes^37–39^. Thus, communities have been used for the complexity and dimensionality reduction of large climate datasets by treating them as coherent sub-systems^14,37,39^. Further, the community detection approach is readily used for the intercomparison of GCMs and their validation against observations^38,40^.

We find that the communities in the *OLR* network correspond to regions with distinct features of ITCZ dynamics that are differentiated by seasonal and interannual variations, topography,and oceanic circulations such as equatorial/coastal upwelling, and ocean currents. We also study the temporal evolution of community structure and network topology on decadal scales. We discover significant variations in the communities encompassing the equatorial Pacific and Atlantic Oceans.

## Results

We use gridded *OLR* data from the $5{th}$ version of spatio-temporal ECMWF Reanalysis (ERA5)^41^ and analyze data for thirty years from 1992 to 2021, with a temporal resolution of 3 hours and a spatial resolution of $1^\circ \times 1^\circ$. See “Materials” for further details. Our study focuses on the spatio-temporal variations of *OLR* over a seasonal scale. Therefore, we neglect temporal scales smaller than 15 days from the time series of *OLR* at all locations and use low-pass filtered *OLR* (denoted as $\widetilde{OLR}$). Moreover, since the migration of the ITCZ in the meridional direction is confined to the tropics^1^, we restrict the spatial domain from $23.5^\circ S$ to $23.5^\circ N$.

**Figure 1:**
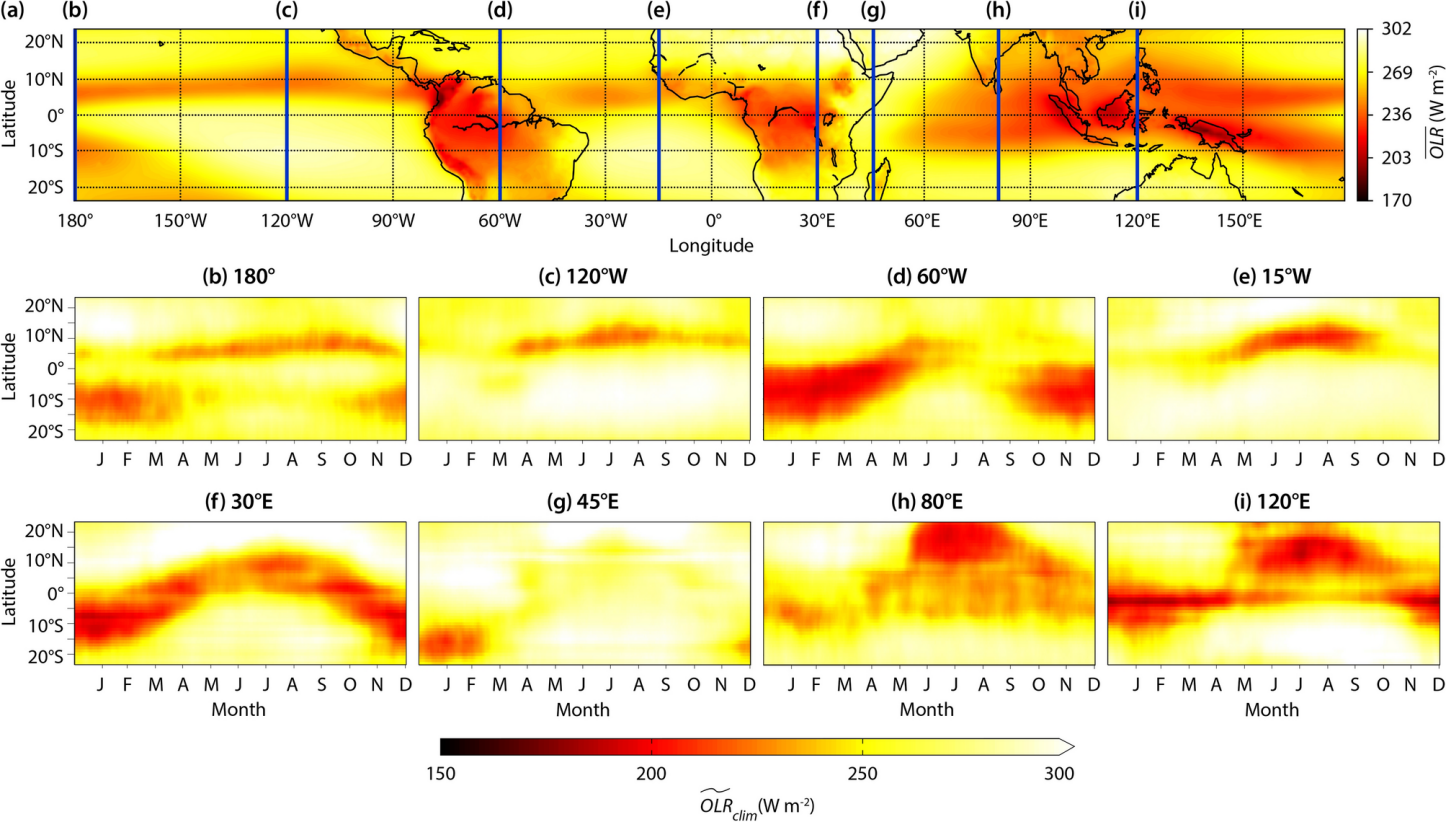
Spatio-temporal dynamics of outgoing longwave radiation (*OLR*) from ERA5 reanalysis data during the period 1992-2021. **(a)** Spatial distribution of time-average outgoing longwave radiation ($\overline{OLR}$). Also shown are the time-latitude diagrams of the low-pass filtered *OLR* climatology ($\widetilde{OLR}_{clim}$) at longitudes marked using vertical lines in **(a)**: **(b)**$180^\circ$, **(c)**$120^\circ W$, **(d)**$60^\circ W$, **(e)**$15^\circ W$, **(f)**$30^\circ E$, **(g)**$45^\circ E$, **(h)**$80^\circ E$, and **(i)**$120^\circ E$. Regions of low $\widetilde{OLR}_{clim}$ ($<240~W/m^2$)^5^, represent the deep clouds associated with the Intertropical Convergence Zone (ITCZ). The spatiotemporal dynamics of the $\widetilde{OLR}_{clim}$ reveal the annual migration patterns of the deep clouds associated with the ITCZ. The characteristics of the ITCZ are sensitive to local conditions such as geography, circulation and convection patterns. As a result, the spatio-temporal dynamics of $\widetilde{OLR}_{clim}$ varies significantly with longitude.

### Spatio-temporal dynamics of $\varvec{OLR}$ in the tropics

The mean structure of the ITCZ is represented by the band of low values of time-averaged *OLR* (denoted as $\overline{OLR}$) girdling the Earth along the equator, as shown in Fig. 1. Time-latitude diagrams of low-pass filtered *OLR* climatology (denoted as $\widetilde{OLR}_{clim}$) at different longitudes (Fig. 1b–i), demonstrate the meridional migration towards the warming hemisphere of deep clouds associated with the ITCZ. Over the western Pacific ($120^\circ E$), central Pacific ($180^\circ$), eastern Pacific ($120^\circ W$) and Atlantic ($15^\circ W$) oceans, the strong easterly trade winds displace warm water slightly northward away from the equator, causing upwelling of cold water from lower depths. As a result, the ITCZ is biased towards the Northern Hemisphere for almost the entire year over these locations^1,2,34,42^. There, the ITCZ is narrow and its amplitude of meridional migration is significantly lower compared to that across $60^\circ W$, $30^\circ E$, and $80^\circ E$. The southeastern Pacific ($120^\circ W$) and the south Atlantic ($15^\circ W$) oceans are not affected by the ITCZ during its annual cycle (Fig. 1c,e).

The central Pacific Ocean is a critical region as the ITCZ transitions from a narrow band in the east to a broader one in the west^1^. Moreover, this is the region where the South Pacific Convergence Zone (SPCZ) branches out in the southeast direction (Fig. 1a). The SPCZ is formed because of the convergence of southeast trade winds over the region of high sea surface temperature (SST) associated with the Indo-Pacific Warm Pool^1,43^. Deep clouds in the Southern Hemisphere over the Pacific Ocean during the austral summer season are associated with the SPCZ.

The underlying surface (land or water) is an important parameter that induces variability in the characteristics of the ITCZ^34^. Over tropical water bodies, the ITCZ is anchored to the region of warm SST^1,3,34^. Due to the high thermal inertia of water, the ITCZ over extended water bodies follows the annual solar heating cycle with a time lag of one or two months. Meanwhile, the thermal inertia of land is negligible compared to water, and as a result, the ITCZ over extended land masses migrates without any time lag.

The effect of the underlying surface on the ITCZ is apparent in the time-latitude diagram of $\widetilde{OLR}_{clim}$ over $60^\circ W$ longitude, as shown in Fig. 1d, which encompasses the continental landmass of South America in the Southern Hemisphere and the Atlantic Ocean in the Northern Hemisphere. We observe that the convection and cloudiness associated with ITCZ are predominantly over South America during the austral spring and summer seasons, while they are relatively weaker over the Atlantic Ocean. The $30^\circ E$ longitude encompasses the extended landmass of Africa. As seen from Fig. 1f, the time-latitude variation of $\widetilde{OLR}_{clim}$ due to the migration of ITCZ along the $30^\circ E$ meridian is nearly sinusoidal. Over this region, the ITCZ follows the solar heating cycle with negligible time lag^1^.

From Fig. 1g, we observe that the ITCZ exhibits an interesting behavior along the $45^\circ E$ meridian. While the coastal upwelling suppresses the ITCZ during the northern spring and summer seasons^2^, the landmass of the island of Madagascar supports the ITCZ during the southern summer season. Due to coastal upwelling, the ITCZ over landmasses is disconnected from its counterpart over extended water bodies^1,2^ (Fig. 1a).

The $80^\circ E$ longitude also covers both land and ocean, encompassing the Indian peninsula north of $8^\circ N$ and the Indian Ocean south of it. As seen from Fig. 1h, from January to March, the ITCZ along the $80^\circ E$ longitude is over the Indian Ocean. Beyond March, it starts propagating north towards the Indian subcontinent, reaching it in June, which leads to the onset of the Asian Summer Monsoon (ASM)^3^. ASM, in turn, enhances the convection over the region, splitting the ITCZ into two zones, one over the Indian peninsula and the other over the Indian Ocean^1^. The northern ITCZ is notably more robust and consistent than the southern one, and exhibits sinusoidal behaviour. Interested readers can refer to^1^ and^2^ for a detailed review on the characteristics of the ITCZ over different regions.

From Fig. 1, we conclude that the dynamics of the $\widetilde{OLR}_{clim}$ driven by the ITCZ’s migration, convection, and cloudiness is strongly heterogeneous and exhibits high spatial variability. Therefore, it is crucial to classify the tropics based on the characteristics of the ITCZ.

### Climate classification using community detection

We perform the climate classification of the tropics from the perspective of complex networks. This approach enables the incorporation of teleconnections and interactions in climate classification. Unravelling teleconnections patterns in the climate system is essential for understanding the underlying physical mechanisms and forecasting anomalous climate events such as heatwaves, droughts, and extreme rainfall^11,16,44^. Here, we are interested in regional and long-range teleconnections arising due to variability in the spatio-temporal dynamics of the $\widetilde{OLR}$, driven primarily by the ITCZ. We construct a functional network with nodes representing different geographical locations. Links are then based on the magnitude and significance of the correlation between the $\widetilde{OLR}$ dynamics of each pair of nodes. The method for constructing the $\widetilde{OLR}$ network is discussed in detail in the “Methods” section.

We classify the tropical climate by partitioning the $\widetilde{OLR}$ network into non-overlapping groups of nodes, where nodes within a group are densely connected while connections between nodes belonging to separate groups are sparse^8^. Such partitioned groups based on the density of connections are referred to as communities and are obtained by community detection algorithms^8,45^. Here, we use the Louvain algorithm^36^, a modularity-optimization algorithm, to identify communities in the $\widetilde{OLR}$ network. Further details are provided in the “Methods” section. In addition to climate classification, complex network analysis also enables the quantification of coherence in the climate dynamics of different communities via estimating measures such as degree and link density.

### Community structure of the $\varvec{\widetilde{OLR}}$ network

**Figure 2:**
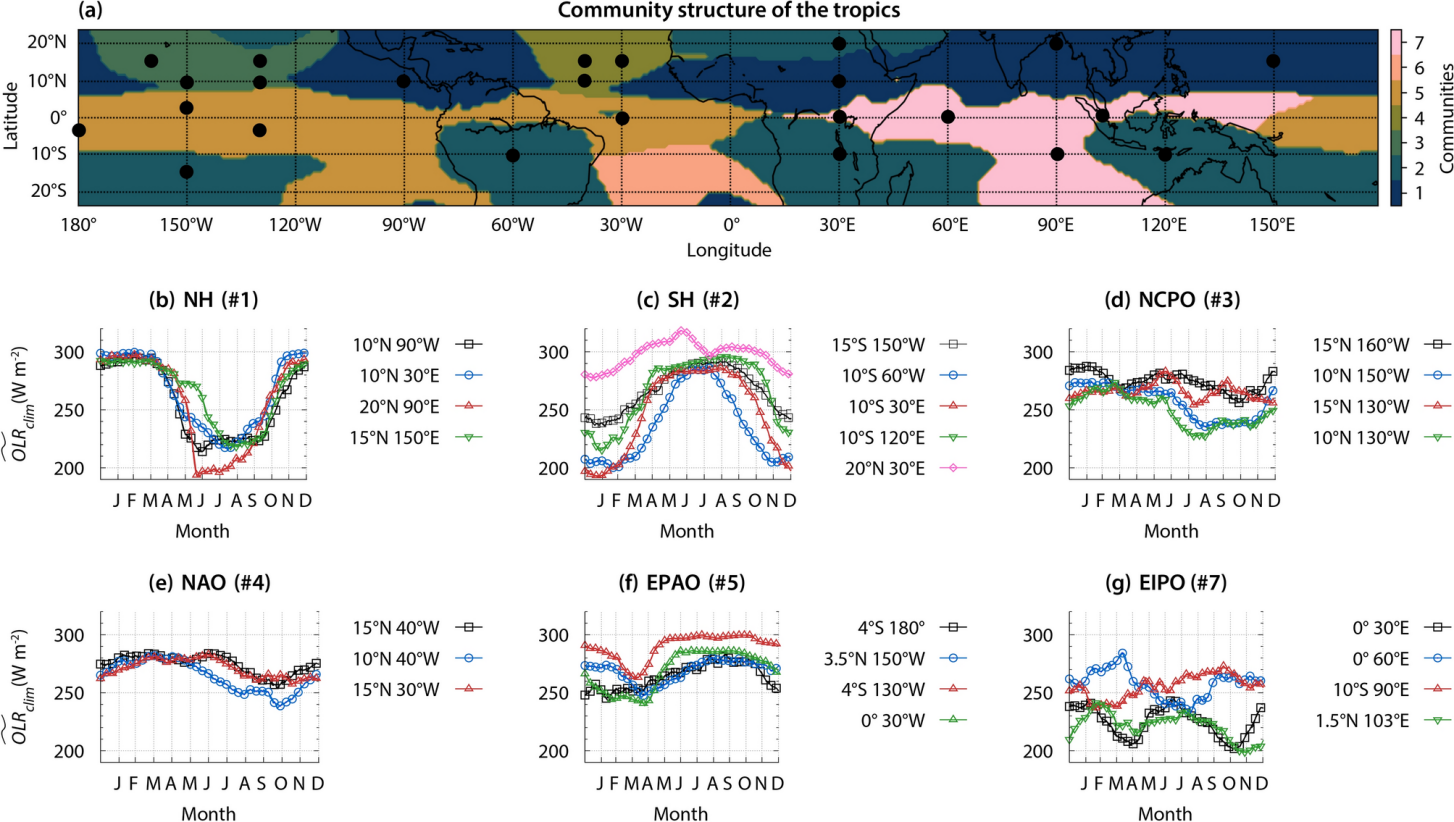
Communities in the $\widetilde{OLR}$ network and time histories of $\widetilde{OLR}_{clim}$for representative locations within a community.** (a)** The community detection algorithm divides the $\widetilde{OLR}$ network into seven communities. These communities reveal the spatio-temporal dynamics of the $\widetilde{OLR}$, driven by the ITCZ’s migration and evolution in an annual cycle. Moreover, these communities divide the tropics into regions of distinct $\widetilde{OLR}$ characteristics. These characteristics are described in **(b–g)** via annual variation of $\widetilde{OLR}_{clim}$ at several representative locations within the communities.

As shown in Fig. 2a, communities identified in the $\widetilde{OLR}$ network enable us to classify the tropics into seven regions. We have assigned a nomenclature (Table 1) to communities according to the region they encompass. Based on their geographical locations, communities can be broadly divided into three categories. Communities NH, NCPO, and NAO are in the Northern Hemisphere, EPAO and EIPO are associated primarily with the equatorial region, and communities SH and SAO are mainly in the Southern Hemisphere. Next, we elucidate the physical interpretation of communities in the $\widetilde{OLR}$ network.

Figure 2b shows the annual variation of $\widetilde{OLR}_{clim}$ (i.e. the climatological average of $\widetilde{OLR}$ ) at four locations marked using the black-filled circles in the NH community (Fig. 2a). As mentioned earlier, the ITCZ is associated with deep convective clouds, identified by monthly averaged $OLR<~240 ~W/m^2$^5^. We observe that, despite the significant geographical distance between these four locations (Fig. 2b), the $\widetilde{OLR}_{clim}$ appears to be synchronized. Deep clouds associated with the ITCZ are observed from May to October over landmasses. In contrast, we uncover that the ITCZ arrives in July at the point over the western Pacific Ocean ($15^\circ N~150^\circ E$). The lag of approximately two months is due to water’s higher thermal inertia than land^1^. We conclude that this community could be interpreted as the Northern Hemisphere region affected by the ITCZ during the northern spring and summer seasons.

**Table 1:** Communities in the $\widetilde{OLR}$ network: nomenclature of the communities, their abbreviations, and link density ($\rho$).

Community	Name	$\rho$
1	Northern hemisphere (NH)	0.423
2	Southern hemisphere (SH)	0.266
3	Northern central pacific ocean (NCPO)	0.235
4	Northern atlantic ocean (NAO)	0.305
5	Equatorial pacific and atlantic oceans (EPAO)	0.137
6	Southern atlantic ocean (SAO)	0.404
7	Equatorial Indian and pacific oceans (EIPO)	0.099

Meanwhile, in the SH community, the time histories of $\widetilde{OLR}_{clim}$ are out-of-phase with respect to those in the NH community (Fig. 2c). The deep clouds of the ITCZ are observed from October to April over landmasses and from December to March over water bodies. Therefore, we interpret the SH community as regions in the tropics that are affected by the ITCZ during the southern spring and summer seasons. Note that deep convective clouds over the southwest Pacific Ocean are due to the SPCZ, which branches out from the maritime continent in the southeast direction.

The point, $20^\circ N~30^\circ E$, is in the Sahara desert, which acts like a barrier to the ITCZ, restricting its excursion beyond approximately $15^\circ N$^46^. Therefore, variations in $\widetilde{OLR}_{clim}$ over this region are not due to the ITCZ. Rather, they are due to seasonal variations in surface temperature. The annual variation of $\widetilde{OLR}_{clim}$ over the Sahara desert is in phase and well correlated with those in the Southern Hemisphere. As a result, these distinct regions are well connected and clustered together in one community. However, the physical mechanisms over the Sahara desert and other regions in the SH community are different. Therefore, we remove connections associated with the Sahara desert region while analyzing the intra-community connections. The effect of removing the connections of the Sahara desert region is discussed in the Supplementary Information.

Over the central Pacific Ocean, the ITCZ is displaced slightly north due to equatorial upwelling (Fig. 1). Figure 2d shows that this section of the ITCZ is observed along the $10^\circ N$ latitude in the NCPO community during the northern summer season. The ITCZ over this region is relatively narrow and the amplitude of meridional migration is low as it stays between $5^\circ N$ and $12^\circ N$ throughout the annual cycle (Fig. 1a,b)^1^. The restricted ITCZ is due to the weakening of convection caused by a sharp negative heating gradient in the meridional direction towards the north. As a result, deep clouds associated with the ITCZ are not identified along the $15^\circ N$ latitude and further north in the NCPO community. The NAO community exhibits a similar behaviour (Fig. 2e). We observe the ITCZ along the $10^\circ N$ latitude for a relatively shorter time in the months of October and November, while further north, the convection resulting in deep clouds associated with the ITCZ is suppressed.

The EPAO community encompasses the equatorial central Pacific, eastern Pacific, and Atlantic oceans, which are associated with equatorial upwelling that suppresses the convection and formation of deep clouds of the ITCZ^2,34,42^. In general, the $\widetilde{OLR}_{clim}$ does not go below the threshold for deep clouds ($240~W/m^2$) over several locations in this community (Fig. 2f), confirming the suppression of the ITCZ. This community also encompasses the southeastern Pacific Ocean, which has unusually lower SST due to the Humboldt Current System^47^. The cooling effect in this system is due to coastal upwelling along the west coast of South America. The low SST suppresses the organized convection. As a result, this region is devoid of the ITCZ.

As seen from Fig. 2g, the annual variation of $\widetilde{OLR}_{clim}$ across the EIPO community is non-uniform. Such behaviour indicates that the spatio-temporal dynamics of the ITCZ changes drastically within this community. Over western Africa ($0^{\circ }~30^\circ E$), the $\widetilde{OLR}_{clim}$ dips twice, indicating that the ITCZ crosses this point twice during its annual migration cycle. First time while moving north from March to May and a second time while moving south from September to December. A similar behaviour, with a small phase shift, is observed at $1.5^{\circ } N~103^{\circ }E$. Meanwhile, over $0^\circ ~60^\circ E$ and $10^\circ S~90^\circ E$, deep clouds associated with the ITCZ are observed only once, approximately from June to September and February to April, respectively. Due to the lower heat capacity of land compared to that over extended water bodies, the convection is more robust over land. Consequently, the clouds of the ITCZ are deeper and $\widetilde{OLR}_{clim}$ is lower over land than extended water bodies.

### Network measures of communities

Next, we explore the structure of connections within the four largest communities in the $\widetilde{OLR}$ network (NH, SH, EPAO, EPIO). The intra-community degree ($k^c$) of a node is the number of connections it has with nodes within the community. It is defined as $k^c_i=\sum _{j=1}^{N^c} A^c_{ij}$, where $N^c$ is the number of nodes in the community and $\textbf{A}^c$ is the intra-community adjacency matrix of size $N^c\times N^c$. Based on $k^c$, we calculate the link density $\rho =\frac{\sum _{i=1}^{N^c}k^c_i/2}{N^c(N^c-1)/2}$, which is the ratio of the number of actual intra-community links to the total number of potential intra-community links. Link densities for the seven communities are listed in Table 1.

**Figure 3:**
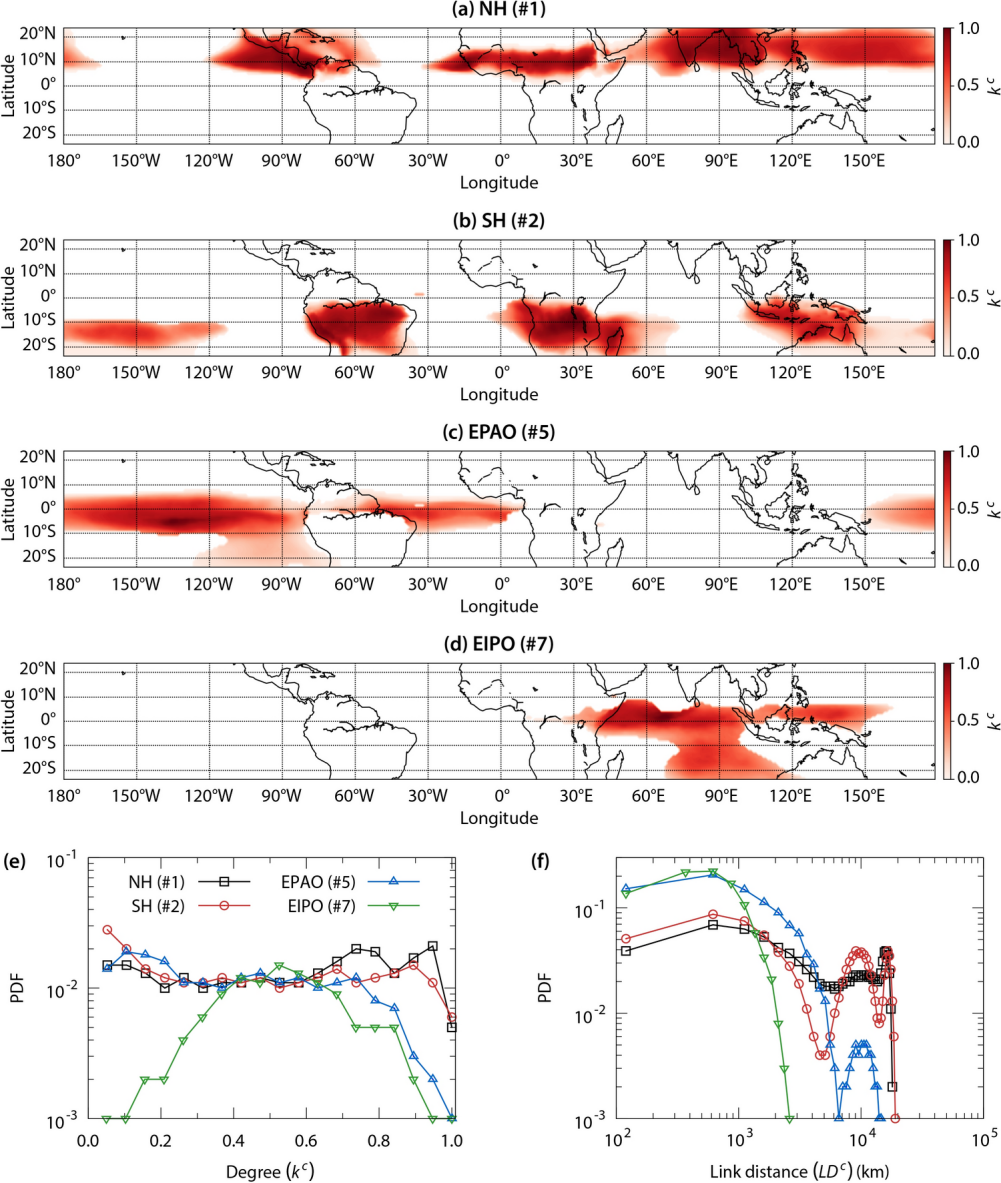
Network measures of communities in the $\widetilde{OLR}$ network. Spatial distribution of intra-community degree ($k^c$) for **(a)** the Northern Hemisphere community (NH), **(b)** the Southern Hemisphere community (SH), **(c)** Equatorial Pacific and Atlantic Oceans community (EPAO), and the **(d)** Equatorial Indian and Pacific Oceans community (EIPO). Shown in **(e)** are the PDFs of $k^c$ for these communities. $k^c$ is normalized using the maximum value for the community. $k^c$ represents the number of direct connections of a node within the community. In the present network, a high value of $k^c$ means that temporal dynamics of $\widetilde{OLR}$ at the corresponding location correlates well with many community locations. Also shown in **(f)** are the PDFs of the link distance ($LD^c$) for communities. The link distance ($LD^c$) is the geographical distance between two connected nodes in the community. Please note that we remove connections associated with the Sahara desert region while analyzing the intra-community connections.

Figures 3a,b show the spatial distribution of $k^c$ for the NH and SH communities, respectively. We have normalized $k^c$ using the maximum value for the community. As expected, $k^c$ is higher over landmasses than over water. For the NH community, most nodes have medium to high degrees. This is also reflected in the probability distribution of $k^c$ (Fig. 3e). The peak in the distribution is towards its right tail. As a result, this community has a high link density ($\rho =0.423$); in fact, it is the highest among all communities. In contrast, the PDF of $k^c$ for the SH community reveals that, unlike the NH community, it has more low-degree nodes, which results in a lower link density ($\rho =0.266$). As seen from Fig. 3b, many low degree nodes are over the western and central southern Pacific Ocean due to non-coherent spatio-temporal $\widetilde{OLR}$ dynamics.

The link densities of intra-community connections of the EPAO and EIPO communities are $\rho =0.137$ and $\rho =0.099$, respectively. We observe from Figs. 3c for EPAO and 3d for EIPO that these communities have sparse connections. Moreover, peaks in the PDFs of $k^c$ for both communities are at low values (Fig. 3e). Unlike NH and SH communities, the decay from the peak of $k^c$ is monotonic. The sparsity in connections within these communities is due to non-uniform temporal $\widetilde{OLR}$ dynamics over the water bodies they encompass.

We also explore the spatial scales in a community by investigating the link distance ($LD^c$), which is the geographical distance between its connected nodes. For a pair of connected nodes *i* and *j*, we estimate the link distance $LD^c_{ij}$ as the great-circle distance between their geographical locations using the Haversine formula. Fig. 3f shows the PDFs of link distances within the four largest communities. The PDF of $LD^c$ for the NH community consists of three peaks. The highest peak is at $LD^c\approx 6.4\times 10^2$ km, representing regional connections within this community. The next peak is at $LD^c\approx 1\times 10^4$ km, and these links are between neighboring continents, as well as between the western Pacific Ocean and South Asia, and the western Pacific Ocean and Central America. The second highest peak is at $LD^c\approx 1.6\times 10^4$ km. These are intercontinental connections, for instance, between South Asia and Central America, and between Central Africa and the western Pacific Ocean. The PDF of $LD^c$ for the SH community is qualitatively similar to that for the NH community. The only difference is that the peak at $LD^c\approx 1\times 10^4$ km is slightly higher than at $LD^c\approx 1.6\times 10^4$ km. The peak at $LD^c\approx 1\times 10^4$ km represents links between neighbouring continents, as well as between the central Pacific Ocean and the Maritime continent, and the central Pacific Ocean and South America. Meanwhile, the peak at $LD^c\approx 1.6\times 10^4$ km corresponds to long-range links such as between South America and the Maritime continent, South America and the Pacific Ocean, and southern Africa and the Pacific Ocean. The presence of long-range connections in these two communities are indicative of the large geographical scale of the ITCZ. Moreover, the co-existence of peaks at both short- and long-range links demonstrates the role of the ITCZ in modulating the regional climate and driving teleconnections within these two communities.

For the EPAO community, we observe two peaks in the PDF of $LD^c$. The highest peak, corresponding to $LD^c\approx 6\times 10^2$ km, represents regional connections, while the second peak, at $LD^c\approx 1\times 10^4$ km, refers to connections between the eastern Pacific and Atlantic Oceans. Meanwhile, the majority of connections in the EIPO community are regional connections. The EIPO community encompasses the region that is affected by the convective activities associated with the Boreal Summer Intraseasonal Oscillation (BSISO) and Madden-Julian oscillation (MJO)^48^. These convective oscillations originate in the central Indian Ocean and contribute significantly towards the variability in the $\widetilde{OLR}$ dynamics^48^. As a result, the connectivity in the EIPO community is sparse and short-range.

### Interdecadal evolution of the community structure of the $\varvec{\widetilde{OLR}}$ network

Next, we explore the temporal evolution of the community structure of the $\widetilde{OLR}$ network on decadal scales. We construct three $\widetilde{OLR}$ networks by dividing the whole data set for thirty years ($1992-2021$) into three non-overlapping sets of 10 years, $1992-2001$, $2002-2011$, and $2012-2021$. For brevity, we will refer to the network constructed for $1992-2001$ as the decade-1 network, $2002-2011$ as the decade-2 network, and $2012-2021$ as the decade-3 network.

**Figure 4:**
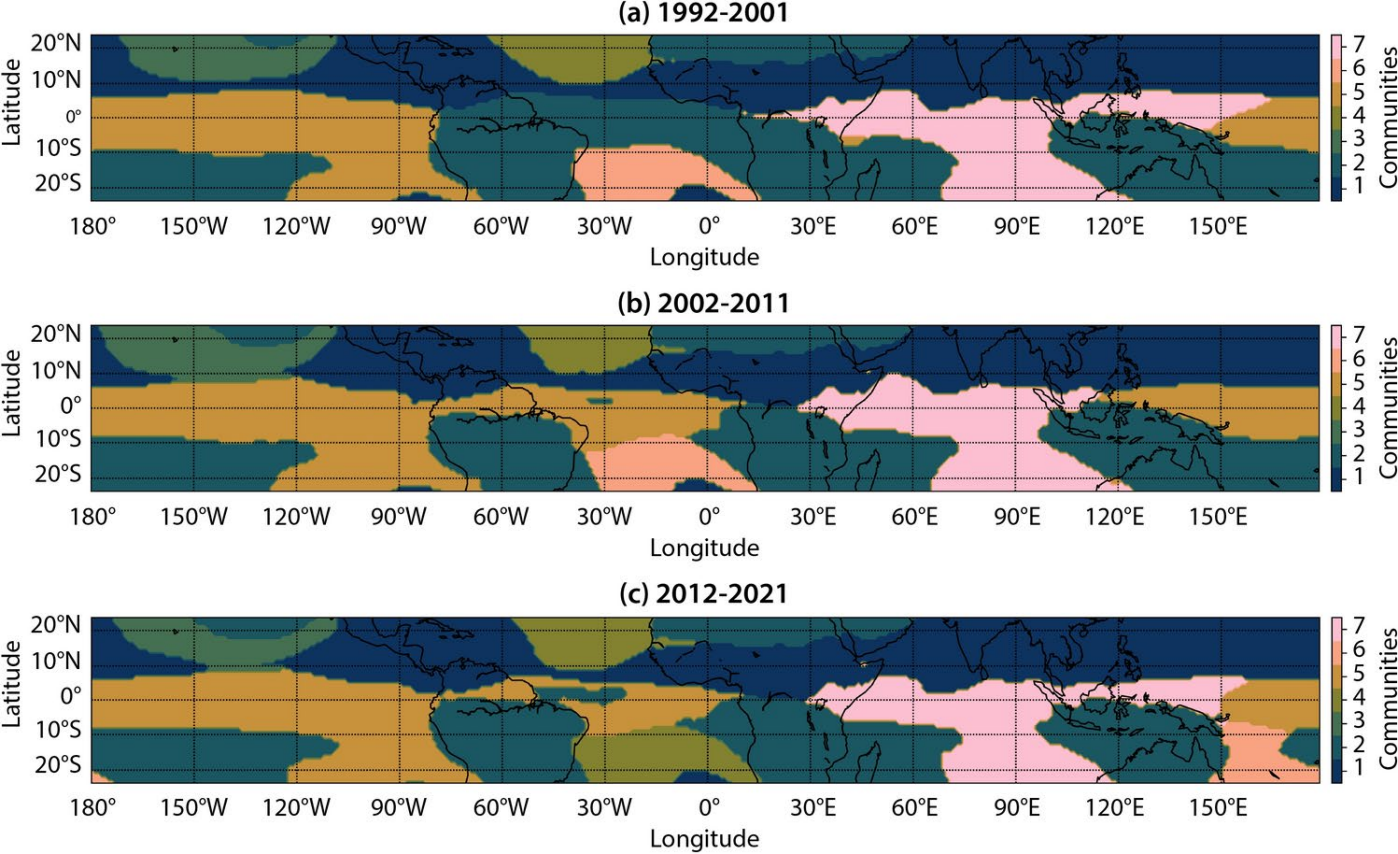
Community structure of decadal networks. Communities in the three decadal $\widetilde{OLR}$ networks: **(a)** decade-1 ($1992-2001$), **(b)** decade-2 ($2002-2011$) and **(c)** decade-3 ($2012-2021$). The NH community ($\#1$) has remained nearly unaltered over the three decades. However, the SH ($\#2$), EPAO ($\#5$), and EPIO ($\#7$) communities exhibit significant variation, especially from the decade-1 to decade-2 time periods. This variation implies that the spatio-temporal dynamics of $\widetilde{OLR}$ over these regions have changed considerably over the past three decades.

The community structures of decade-1, decade-2, and decade-3 $\widetilde{OLR}$ networks shown in Fig. 4 highlight the regions that exhibit significant variation in the multidecadal temporal scale. The structures of NH (1), NCPO (3), NAO (4), and SAO (6) communities appear stable and consistent over the three decades. Most significant variations are observed in the SH (2), EPAO (5), and EIPO (7) communities, especially when comparing decade-1 to decade-2. In decade-1, the equatorial Atlantic Ocean is not a part of the EPAO community, but of the SH community. However, later in decade-2 and decade-3, the equatorial Atlantic Ocean became a part of the EPAO community. Further, in the decade-2 network, the EPAO community extends up to $120^\circ E$, while in decade-1 and decade-3 networks, this community is restricted to $150^\circ E$.

To further understand the reasons behind the changes in the community structure, we investigate the evolution of the underlying connection topology from one decade to the next. We use the Salton cosine-similarity index^49^ to measure the number of common connections of a node across $\widetilde{OLR}$ networks for two successive decades. For a node *i*, the Salton similarity index is estimated as $S_i=\frac{|\Gamma _{i,d}\cap \Gamma _{i,d+1}|}{\sqrt{k_{i,d} \times k_{i,d+1}}}$^49,50^, where $\Gamma _{i,d}$ and $k_{i,d}$ are the set of nodes and total number of nodes that are directly connected to node *i* in decade *d*, respectively. The latter quantity is often referred to as the degree centrality^8^, and for a node *i*, it is defined as $k_i=\sum _{j=1}^{N} A_{ij}$, where *N* is the total number of nodes and $\textbf{A}$ is the adjacency matrix of the network. $S_i=1$ implies that the connectivity pattern of node *i* is exactly the same, while $S_i=0$ means that it has no common connections across two networks. Thus, a low (high) value of $S_i$ indicates that the topology of connections of node *i* across networks of successive decades is significantly different (similar). Here, a significant change in the topology of connections implies a significant variation in the $\widetilde{OLR}$ dynamics. Furthermore, we propose and perform a novel statistical significance test for the Salton similarity index to determine if the difference between network topologies is due to physical mechanisms rather than random variations. A detailed explanation of the significance test can be found in the “Methods” section. We have also studied the effect of increasing the spatial resolution from $1^\circ \times 1^\circ$ to $0.5^\circ \times 0.5^\circ$. As discussed in the Supplementary Information, the spatial distribution of *S* does not change by increasing the spatial resolution. Thus we perform the analysis at $1^\circ \times 1^\circ$.

**Figure 5:**
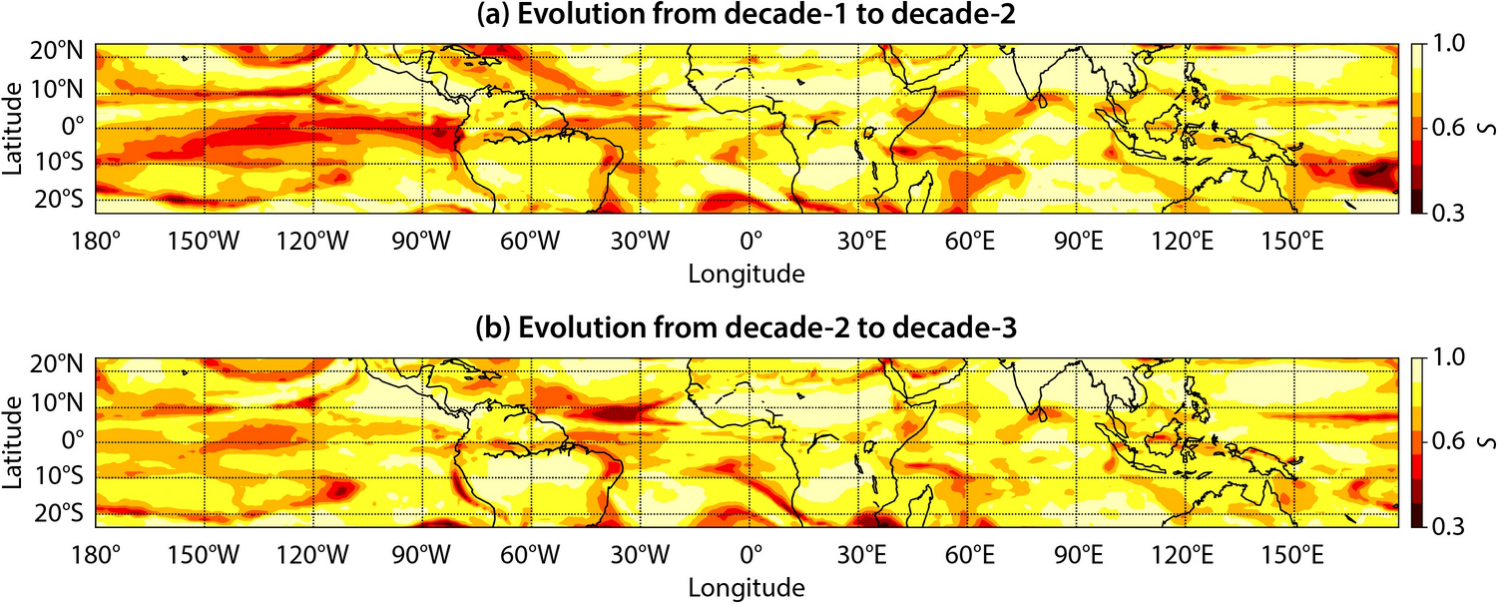
Evolution of network topology from decade-1 to decade-2 and decade-2 to decade-3. Spatial distribution of significant Salton similarity index (*S*) estimated for (**a**) decade-1 and decade-2 networks, and **(b)** decade-2 and decade-3 networks. A low (high) value of *S* implies that the node has a small (large) number of common connections in both networks.

The equatorial central and eastern Pacific and equatorial Atlantic Oceans exhibit low values of *S* across networks corresponding to decade-1 to decade-2 (Fig. 5a), as well as across decade-2 to decade-3 networks (Fig. 5b). Larger regions exhibiting low values of *S* are visible for the evolution from decade-1 to decade-2 compared to decade-2 to decade-3, indicating lesser variability in the $\widetilde{OLR}$ dynamics when evolving from decade-2 to decade-3. Low values of *S* in the equatorial central and eastern Pacific and equatorial western Atlantic Oceans also explain the high variability in the structure of the EPAO community that encompasses these regions. High variability in these regions could be due to natural interdecadal phenomena or to climate change in the past few decades attributable to anthropogenic factors.

One example of a natural intradecadal phenomenon is ENSO, which occurs primarily in the equatorial central and eastern Pacific Ocean. The frequency of warm (El Niño) and cold (La Niña) phases of ENSO drives interdecadal natural variability in this region. On the other hand, high interdecadal variability in the south-western Pacific Ocean (Figs. 4,5) could be attributed to climate change. This region is part of the Pacific Warm Pool, the world’s largest tropical oceanic mass with a SST higher than $28.5^\circ C$. Earlier studies have shown that the Pacific Warm Pool has become warmer and growing in size over the past few decades due to warming caused by anthropogenic factors^51,52^. Therefore, natural variability and climate change might have contributed to altering the characteristics of the ITCZ, which is reflected in our analysis.

The equatorial Atlantic Ocean also exhibits high variability in the $\widetilde{OLR}$ dynamics as indicated by low *S* (Fig. 5) and changes in the community structure (Fig. 4). This region is part of the Atlantic Warm Pool (AWP)^53^, also known as the Western Hemisphere Warm Pool^54^, which is the second-largest tropical warm pool on Earth. The AWP is a critical region since it lies in the development area of Atlantic tropical cyclones. Variability in the AWP affects their frequency and strength. A well-known source of natural variability in the AWP is the Atlantic Multidecadal Oscillation (AMO)^53^, which alters its size and SST in a time scale of several decades. Another phenomenon that could be contributing to the changes in the network measures over the equatorial Atlantic Ocean is the inter-decadal variability in the patterns of Atlantic Niño^55^. The Atlantic Niño is associated with warm SST anomalies in the central and eastern Equatorial Atlantic Ocean. Since 2000, the central component of the Atlantic Niño is strengthening compared to the eastern component^56^. This phenomenon could be contributing to the separation of the equatorial Atlantic Ocean from the SH community in the decade-1 network and merging with the EPAO community in the decade-2 network (Fig. 4a,b).

In summary, this analysis can identify regions that exhibit high variability on decadal scales. However, it is critical for ongoing research to identify whether the interdecadal variability observed here is due to natural phenomena or anthropogenic factors. Another open yet critical question is whether anthropogenic factors compound the natural interdecadal and intradecadal phenomena.

## Discussion

In the present study, we propose a climate classification of the tropics based on the spatio-temporal dynamics of the ITCZ. We use the framework of complex networks to analyze the outgoing longwave radiation (*OLR*), which is a good proxy for convection and deep clouds in the ITCZ^3,5^. A complex network is constructed by establishing links between pairs of geographical locations when the temporal dynamics of low-pass filtered *OLR* ($\widetilde{OLR}$) are significantly correlated based on the Pearson correlation coefficient. This way, we achieve a novel classification of the tropics by identifying seven communities in the $\widetilde{OLR}$ network using the Louvain algorithm^36^. Communities are non-overlapping groups, where nodes within a group are densely connected, and nodes between different groups are sparsely connected. Here, we show that communities represent regions with distinct ITCZ characteristics, thereby classifying the tropics based on their dominant dynamics. The classification proposed here gives additional insights to enhance the physical understanding of the spatio-temporal dynamics of the ITCZ over different tropical regions and its role in modulating the regional climate and the teleconnection patterns. Mainly, we find that:

(i) The two largest communities, the Northern (NH) and Southern Hemisphere (SH) represent regions affected by the ITCZ during the northern and southern summer seasons. These communities are associated with coherent $\widetilde{OLR}$ dynamics driven by the ITCZ migration. Due to the global-scale structure of the ITCZ, these communities have a large amount of long-range intercontinental teleconnections. Due to coherent $\widetilde{OLR}$ dynamics over these communities, a reduced-order model governing the dynamics in these regions could be developed and incorporated to speed up simulation and prediction models.
(ii) The Equatorial Pacific and Atlantic Oceans (EPAO) and Equatorial Indian and Pacific Oceans (EIPO) communities encompass extended water bodies associated with incoherent $\widetilde{OLR}$ dynamics. As a result, these communities have sparse connections, most of which are short-ranged or regional. We observe that the EPAO community is devoid of deep convective clouds of the ITCZ. Strong easterly trade winds displace warm water from the equator towards the north, leading to equatorial upwelling that suppresses the convection. Further, over the southeast Pacific Ocean this community is devoid of the ITCZ because of coastal upwelling along the east coast of South America. There, coastal upwelling is associated with a larger Humboldt Current System. For the EIPO community, the high variability in $\widetilde{OLR}$ dynamics can be attributed to the convective activities in the Indian and Pacific Oceans due to the BSISO and MJO^48^.
(iii) During different ENSO phases, the region associated with the EPAO community experiences fluctuations in wind and SST, which results in extreme weather events such as excess rainfall or droughts across tropics and sub-tropics. Further, the extent and depth of the Pacific Warm Pool fluctuates with ENSO, consequently modulating the size and strength of the South Pacific Convergence Zone. Therefore, ENSO significantly impacts the topology of climate networks^21^. Studying the variation of the community structure of the $\widetilde{OLR}$ network during ENSO events can help develop precursors for extreme weather events over several regions in the tropics and subtropics.

The improved understanding of the ITCZ dynamics gained with our climate classification for the tropics can enhance weather forecasting models, develop regional reduced-order models, validate and improve future global circulation models and monitor climate change.

We also study the temporal evolution of the $\widetilde{OLR}$ network and the corresponding community structure on decadal scales. We uncover that the EPAO community exhibits significant variations on interdecadal scales from 1992 to 2021. This community has the highest variability in the central and eastern Pacific oceans, the Pacific Warm Pool, and the Atlantic Warm Pool. Attributing this multidecadal variability to a single source or phenomenon is difficult. The variability could be due to natural interdecadal oscillations such as the Atlantic Multidecadal Oscillation (AMO), intradecadal oscillations such as ENSO, climate change caused by anthropogenic factors, or the compounding effect of climate change on natural phenomena. These questions remain open for future research.

Networks constructed using climatological variables have some small-world-like architecture^12^, i.e., they borrow characteristics of both regular lattice and random networks. A small-world network provides stability and contains sufficient long-range links, like in a random network, which enables the transfer of perturbations across the network quickly and efficiently. We explore the role of the ITCZ in enabling such architecture. We find that, due to the presence of distinct communities, the *OLR* network has high clustering. Further, the large spatial structure of the ITCZ enables long-range connections in the climate network. These characteristics result in the *OLR* network exhibiting some small-world architecture. This suggests that the spatio-temporal dynamics of the ITCZ provides stability to the climate system against perturbations or fluctuations by spreading them uniformly and efficiently across the globe via long-range teleconnections. Perturbations could be extreme events such as strong ENSO phases, droughts, excessive rainfall, heat waves, etc.

This study uses linear interactions estimated via Pearson correlation coefficient to represent climate connections and teleconnections. However, extreme weather events such as excessive rainfall are usually caused by teleconnections associated with nonlinear interactions^11,57^. In the future, we will extend the climate classification method proposed here for nonlinear interactions estimated by statistical similarity measures such as conditional mutual information or event synchronization. A climate classification based on non-linear interactions will help identify regions susceptible to extreme weather events. Further, multidecadal analysis will highlight regions where extreme weather events are increasing due to climate change caused by anthropological factors. The primary goal of the present study is to demonstrate the ability of complex networks and community detection to perform meaningful climate classification. The Louvain algorithm has a potential drawback in that it could generate communities that may be internally disconnected. The Leiden algorithm can be utilized in such scenarios, which guarantees internally connected communities^58^. Given that the Earth’s climate has high complexity, one should be careful in deriving physical mechanisms from network analysis and community detection. Future research should incorporate interactions occurring over a wide range of spatiotemporal scales and between different subsystems (atmosphere-land and atmosphere-ocean) to further improve our understanding. Another potential application of the approach presented here could be to study spatiotemporal patterns and teleconnections associated with extreme weather events using anomalies.

## Materials

We employ gridded *OLR* data from the $5^{th}$ version of the spatio-temporal ECMWF Reanalysis (ERA5)^41^. Data for thirty years (1992-2021) with a temporal resolution of 3 hours is considered. Since the migration of the ITCZ occurs within the tropics, we restrict the spatial domain from $23.5^\circ N$ to $23.5^\circ S$. The grid points are equally spaced with a spatial resolution of $1^\circ \times 1^\circ$, resulting in a total number of $N=17,280$ grid points.

**Figure 6:**
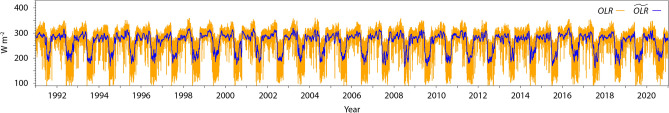
ERA5 reanalysis data sample. Time series of *OLR* and low-pass filtered *OLR* ($$\widetilde{OLR}$$) at $$21^{\circ }N$$$$79^{\circ }E$$. The high-frequency oscillations are suppressed in $$\widetilde{OLR}$$ while it retains the seasonal variation due to migration of the ITCZ.

Figure 6 shows the time series of *OLR* at $21^{\circ }N~79^{\circ }E$ from $2012-2021$. The time series primarily consists of two states, low and high *OLR*. The low *OLR* state corresponds to the presence of deep clouds associated with ITCZ, while the high *OLR* state corresponds to their absence. Moreover, the ITCZ state is associated with relatively large amplitude high-frequency oscillations, primarily due to temperature variations from day to night. In the present study, we are concerned with the seasonal variation of the ITCZ. Therefore, we neglect the shorter-scale temporal variations and use low-pass filtered *OLR* (denoted as $\widetilde{OLR}$), i.e., high-frequency oscillations are suppressed in the $\widetilde{OLR}$, whereas it retains the annual variations associated with the meridional migration of the ITCZ. In fact, the variations are more apparent in $\widetilde{OLR}$ compared to *OLR*. The $\widetilde{OLR}$ is estimated by performing short-time moving window averaging:

1 $$\begin{aligned} \widetilde{OLR}(t,lat,lon)=\frac{1}{t_w}\sum _{t-t_w/2}^{t+t_w/2}OLR(t,lat,lon) dt. \end{aligned}$$

We consider $t_w=15$ days. The criteria of $\widetilde{OLR}<240~W/m^2$ is used to identify deep convective clouds in the ITCZ^5^.

## Methods

### $\varvec{\widetilde{OLR}}$ network construction

We consider the discrete grid points on the spatial domain as the network nodes ($N=17,280$). Then, a link between two nodes *i* and *j* is established based on the magnitude and significance of the Pearson correlation coefficient ($R_{ij}$) between their $\widetilde{OLR}$ time series. Pearson correlation, which is a linear measure, has been successfully applied in previous studies for constructing climate networks^4,12,15,17,59^. We construct the correlation matrix ($\textbf{R}$) of size $N \times N$, whose elements ($R_{ij}$) are estimated as:

2 $$\begin{aligned} R_{ij} = \frac{ \mathbb {E}\left[ \bigl (\widetilde{OLR}_i-\mathbb {E}[\widetilde{OLR}_i]\bigr ) \bigl (\widetilde{OLR}_j-\mathbb {E}[\widetilde{OLR}_j]\bigr ) \right] }{\sqrt{\mathbb {E}\left[ \bigl (\widetilde{OLR}_i-\mathbb {E}[\widetilde{OLR}_i]\bigr )^2\right] } \sqrt{\mathbb {E}\left[ \bigl (\widetilde{OLR}_j-\mathbb {E}[\widetilde{OLR}_j]\bigr )^2\right] }}, \end{aligned}$$

where $\mathbb {E[\cdot ]}$ denotes the expected value of a time series. We do not consider time-delayed correlations in our analysis since our objective is to understand and classify the tropical climate based on the dynamics occurring on seasonal time-scales. Next, we threshold the correlation matrix ($\textbf{R}$) and convert it into a binary matrix ($\textbf{A}$), known as the adjacency matrix in network science^8,9^. Elements of $\textbf{A}$ are estimated as:

3 $$\begin{aligned} A_{ij} = {\left\{ \begin{array}{ll} \begin{array}{ll} 1, & \text{ if } R_{ij} \ge \tau \\ 0, & \text{ if } R_{ij} < \tau \end{array} \end{array}\right. }, \end{aligned}$$

where $\tau$ is the threshold for establishing links in the network. We choose $\tau =0.5$ since several earlier studies reported this value of the Pearson correlation coefficient as an appropriate criterion for constructing climate networks^12,14,16,17,21,37^. This threshold provides a good balance between statistical significance and richness in underlying physics. With this thresholding, we get an unweighted and undirected network with a link density of $7\%$.

The degree of node *i*, denoted as $k_i$, is the total number of links it has in the network and is calculated as $k_i=\sum _{j=1}^{N} A_{ij}$. Since our spatial domain is bounded, links in the network could be restricted by the boundary of the domain, altering the degree distribution of the $\widetilde{OLR}$ network^60^. We test the significance of the boundary effects induced by the spatial embedding following Rheinwalt *et al.*, 2012^60^. We find that the degree distribution of the network adjusted for the effect of spatial embedding and the degree distribution of the original network are in very good qualitative agreement, confirming that the boundary effects on the topology of the $\widetilde{OLR}$ network are negligible. We further discuss the effect of spatial embedding in the Supplementary Information. Functional networks, where links are established on the basis of statistical similarity, are prone to biases owing to multiple comparisons. As discussed in the Supplementary Information, methods to correct for biases are highly restrictive^11^ and would prevent a comprehensive climate classification of the tropics. Therefore, we do not adopt multiple testing in this study.

#### Statistical significance test

We test the statistical significance of the Pearson correlation coefficient between two connected nodes ($R_{ij}\ge 0.5$) by performing randomization experiments^12,15,20,21,37^. We construct 1000 surrogates for one node by randomly shuffling the original $\widetilde{OLR}$ time series. Next, we calculate the correlation between the surrogates and the original time series of the other node. We observe that $R_{ij}=0.5$ does not arise “by chance” for any pair of nodes, indicating that all network links are based on statistically significant correlations. In the random shuffling method, we preserve the probability distribution of the time series. We also perform a significance test using the Fourier surrogate method where the power spectrum of the time series is maintained. The surrogate time series is obtained by first performing the Fourier transform of the original time series, followed by randomization of phases and performing the inverse Fourier transform. As a result, the Fourier surrogate method is computationally expensive compared to the random shuffling method. Therefore, we perform the test only on a small number of representative connections across a wide range of correlations and link distances. We find that the original *R* is larger than the $95^{th}$ percentile of the null model constructed with Fourier surrogates, indicating that the correlations still arise due to physical mechanisms and not random coincidence.

### Community detection in the $\varvec{\widetilde{OLR}}$ network

We use the Louvain algorithm^36^ for performing community detection in the $\widetilde{OLR}$ network. This algorithm is based on modularity optimization. Modularity quantifies the quality of the partitioned network, and it represents the difference between the link density of identified communities and randomly connected networks of the same size^8,9^. Modularity is defined as:

4 $$\begin{aligned} \mathscr {H}=\frac{1}{2m}\sum _{c=1}^{n_c}\biggl \{ e_c - \gamma \frac{K_c^2}{2m} \biggr \}, \end{aligned}$$

where $\gamma$ is the resolution parameter, $n_c$ is the number of communities in the network, *m* is the total number of links in the network ($m=\sum _{i=1}^{N}k_i/2$), $e_c$ is the number of intra-community connections in the community *c* ($e_c=\sum _{i=1}^{N^c}k^c_i$), and $K_c$ is the sum of degrees of all the nodes in the community *c* ($K_c=\sum _{i=1}^{N^c}k_i$). The resolution parameter ($\gamma$) governs the size of the communities. Higher $\gamma$ leads to a higher number of communities, while low $\gamma$ leads to fewer communities. The Louvain algorithm is efficient, in terms of computational costs, for detecting communities in large networks. In the present work, we use the Louvain algorithm’s implementation in the Python library NetworkX^61^.

**Figure 7:**
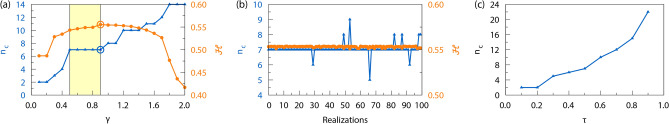
Robustness analysis of the community structure. **(a)** Variation of the number of communities ($n_c$) and modularity ($\mathscr {H}$) with the resolution parameter ($\gamma$). The highlighted region represents the longest plateau in the $n_c~vs~\gamma$ plot. Community detection is carried out using $\gamma =0.9$ since $\mathscr {H}$ is highest at this $\gamma$ within the plateau and overall. $\gamma =0.9$ is marked in the plot. **(b)** The community detection algorithm is run 100 times independently. Most runs identify seven communities in the $\widetilde{OLR}$ network with similar structure and $\mathscr {H}$. **(c)** Sensitivity of $n_c$ to the correlation threshold ($\tau$) for establishing links in the $\widetilde{OLR}$ network.

We determine the appropriate value for $\gamma$ by testing its sensitivity on the community structure^62,63^. The objective is to identify a range of values for which the community structure does not exhibit significant variation. Then, the appropriate $\gamma$ for community detection is selected from this range. Figure 7a shows the variation in the number of communities ($n_c$) and in the modularity of the community structure ($\mathscr {H}$) with $\gamma$. We observe a step-wise increase of $n_c$ with $\gamma$. The first step, for which $0.5\le \gamma \le 0.9$ and $n_c=7$, is the longest in terms of the range of $\gamma$. Hence, we select $\gamma$ from this range. Then, we observe that the modularity is maximum ($\mathscr {H}=0.55$) for $\gamma =0.9$. Further, to establish the convergence of $\gamma$, we perform 100 independent runs of the community detection algorithm on the $\widetilde{OLR}$ network. Figure 7b shows the variations of $n_c$ and $\mathscr {H}$ with 100 realizations. The majority of runs identify seven communities with similar structures and modularities. Therefore, we choose $\gamma =0.9$ for the present study.

The community structure is also sensitive to the correlation threshold for establishing links^14,39^. A small value of the threshold $\tau$ allows links that are not statistically significant, leading to a well-connected network that is less fragmented and has low $n_c$. The increase in $\tau$ leads to the removal of insignificant links, causing fragmentation and rapid increase in $n_c$^14^. We observe this behavior in the $\widetilde{OLR}$ network (Fig. 7c). Therefore, the value of $\tau$ should be a good compromise between the statistical significance of the network and the number of identified communities. We select $\tau =0.5$ because this value implies the statistical significance of the Pearson correlation while giving a meaningful community structure based on actual climatological physics.

### Statistical significance test for Salton similarity index

We assess the significance level of the Salton similarity index (*S*) by developing a null model using a novel method. Salton’s similarity index (*S*) measures the difference between the connectivity and topology of two networks of the same size. Salton similarity index is estimated as $S_i=\frac{|\Gamma _{i,d}\cap \Gamma _{i,d+1}|}{\sqrt{k_{i,d} \times k_{i,d+1}}}$^49,50^, where $\Gamma _{i,d}$ and $k_{i,d}$ are the set of nodes and total number of nodes that are directly connected to node *i* in decade *d*, respectively. A low value of *S* implies that the connectivity of a node in two networks is significantly different, while a high value of *S* suggests it is similar. We explain the significance test by comparing the decadal $\widetilde{OLR}$ networks for periods $2002-2011$ (decade-2) and $2012-2021$ (decade-3). The goal of the test is to assess if the low value of *S*, which indicates a large divergence in the network topology, is because of physical mechanisms instead of random variations in the network topology. Therefore, the null hypothesis is that the variation in the network topology from decade-2 to decade-3 is because of random variations. We construct 1000 surrogate networks by rewiring the connections of the reference network (decade-2). The rewiring is done by randomly shuffling the connections of certain nodes and modifying the topology of the reference network while keeping the link density constant. Nodes that are rewired are selected randomly. However, their number equals the number of nodes whose connection topology exhibits significant variation ($S<0.6$) from one decade to the next. We obtain the null distribution by estimating *S* between the original and surrogate networks. We consider *S* estimated between decade-2 and decade-3 networks to be significant if it is less than the $5^{th}$ percentile of the null distribution. Values in the left tail of the distribution are considered because a low *S* implies a significant variation in the network topology across decades.

## Supplementary Information

Supplementary Information.

## Data Availability

The outgoing longwave radiation reanalysis data that supports the findings of this study is publicly available online: ERA5 Reanalysis data^41^ (https://doi.org/10.24381/cds.adbb2d47).

## Code availability

The codes used in this study are available at https://doi.org/10.5281/zenodo.8176657
