# Supplementary material for: Community structure of tropics emerging from spatio-temporal variations in the Intertropical Convergence Zone dynamics
Source: Sci Rep. 2024 Oct 18;14:24463. doi: 10.1038/s41598-024-73872-0 (PMC11489660; doi:10.1038/s41598-024-73872-0)
Supplement: Supplementary file 1 — Supplementary Information. [file 41598_2024_73872_MOESM1_ESM.pdf]

Community structure of tropics emerging from spatio-temporal variations in the Intertropical Convergence Zone dynamics

Supplementary Information

Gaurav Chopra, Vishnu R. Unni, P. Venkatesan, Sara M. Vallejo-Bernal, Norbert Marwan*, Jürgen Kurths, R. I. Sujith
Cooresponding author e-mail: marwan@pik-potsdam.de

Topology of the $\widetilde{OLR}$ network

Degree

In a complex network, the degree of the node i (k_i) is defined as the number of nodes with which it has a direct connection and is a fundamental centrality measure. In an unweighted and undirected network, considered here, the degree is defined as $k_i = \sum_{j=1}^{N} A_{ij}$, where N is the total number of nodes in the network and $\mathbf{A}$ is the adjacency matrix that encodes the topology of the network [Boccaletti et al., 2006]. In this study, we have established connections based on Pearson's correlation coefficient. Therefore, high k_i means that the temporal $\widetilde{OLR}$ dynamics of a node i is well correlated with that for a large number of nodes and *vice versa*.

As seen from figure 1, regions that are affected by the ITCZ during its annual migration cycle have a high degree. These regions are associated with several monsoon systems, such as the Asian summer, north Australian, African, and American monsoons that depend on the precipitation of the ITCZ. Figure 1 exhibits an important finding: k is relatively lower in a narrow zone along the equator, while it is high in certain regions north and south of this zone. Low k in this zone reveals that the ITCZ exhibits high variability over the equatorial region. k is higher over land and coastal regions than extended oceans and seas. This pattern indicates that the spatio-temporal dynamics of $\widetilde{OLR}$ due to migration of the ITCZ over land is more coherent than over water bodies.

Average clustering and path length

The average clustering coefficient (C) is an important metric that helps identify if the network topology is regular, random, or small-world Watts and Strogatz [1998], Humphries and Gurney [2008]. The clustering coefficient of a node represents the number of its neighbours that are also interconnected Watts and Strogatz [1998], and it quantifies segregation in the network van Diessen et al. [2014]. The average clustering coefficient (C) is the mean clustering coefficient of all the nodes, and it is defined as $C = \frac{1}{N}\sum_{i=1}^{N}\frac{2E_i}{k_i(k_i-1)}$, where E_i is the number of connections between neighbours of node i. Functional networks based on climatological variables share some properties of small-world networks Tsonis and Roebber [2004]. Small-world networks are associated with a high degree of clustering along with the presence of long-range links, making them efficient in distributing information uniformly across the network.

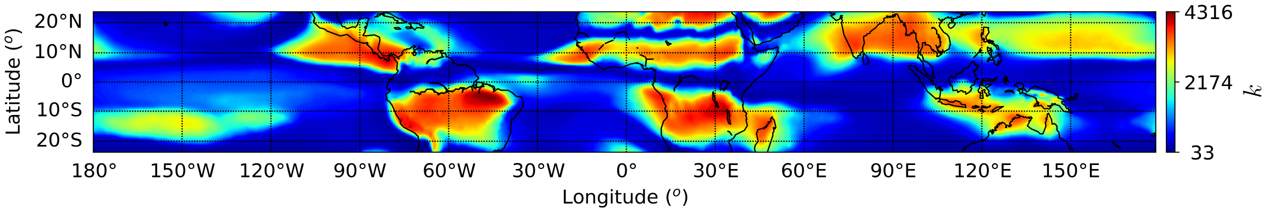

Figure 1: **Degree centrality (k) of the $\widetilde{OLR}$ network:** degree centrality of a node represents the total number of links of the node. In the present network, a high value of k suggests that the $\widetilde{OLR}$ time history of the node is well correlated with that of a large number of other nodes. Regions affected by the seasonal migration of the ITCZ have a relatively higher degree, particularly regions associated with monsoon systems. In general, extended landmasses and coastal regions have a higher degree compared to extended seas and oceans, which brings out the land-water contrast in the temporal dynamics of the $\widetilde{OLR}$.

The spatio-temporal dynamics of the ITCZ results in an $\widetilde{OLR}$ network that has a high degree of clustering ($C = 0.684$) and a modular community structure (see figure 2). Further, the large spatial structure of the ITCZ promotes long-range teleconnections in the climate, which results in long-range links in the $\widetilde{OLR}$ network (see figure 3 (f)) [Donges et al., 2009, Boers et al., 2014]. Consequently, the topology of the $\widetilde{OLR}$ network exhibits some small-world characteristics, suggesting that the spatio-temporal dynamics of the ITCZ plays an important role in enabling such characteristics particularly highly efficient energy transfer mechanism. Steps followed to determine the small-worldness of the $\widetilde{OLR}$ network are discussed in the section "Materials and Methods". In functional climate networks, small-world characteristics enable the efficient (uniform and swift) distribution of energy across the globe [Tsonis and Roebber, 2004]. Thus stabilizing the climate against climatic fluctuations and preventing prolonged extreme events such as droughts, excess rainfall, heatwaves, El Niño–southern oscillations, etc. [Tsonis and Roebber, 2004, Tsonis and Swanson, 2008]

Boundary effects due to spatial embedding

The spatial domain of our study is bounded by artificially induced boundaries, which makes the spatial distribution of network measures susceptible to the effects of spatial embedding. Boundaries destroy links with nodes that are outside of the spatial domain. Moreover, the reduction of links is non-uniform. Nodes near the boundaries suffer higher reductions than those in the interior [Rheinwalt et al., 2012, Gupta et al., 2021]. This could lead to incorrect interpretation of network measures. Therefore, testing for boundary effects on the network measures is critical. We test the effect of spatial embedding on the degree (k) of the $\widetilde{OLR}$ network according to the procedure proposed in Rheinwalt et al. [2012]. In this procedure, we construct 1000 surrogate random networks with the same link probability based on spatial link distance as the original network. Such random networks are known as spatially embedded random networks. To adjust for the boundary effects, we subtract the degree estimated from the mean of 1000 surrogates from the original degree.

The activity in $\widetilde{OLR}$ due to the migration of the ITCZ is restricted to the tropics. Connections between the nodes in the spatial domain considered here and the nodes outside will be negligible. Therefore, we expect boundary effect corrections will not significantly influence the network topology. The effect will be quantitative. Indeed, as seen from figure 2, the original and modified degrees are in excellent qualitative agreement. Hence, we discuss both the qualitative and quantitative aspects of the original degree in this work.

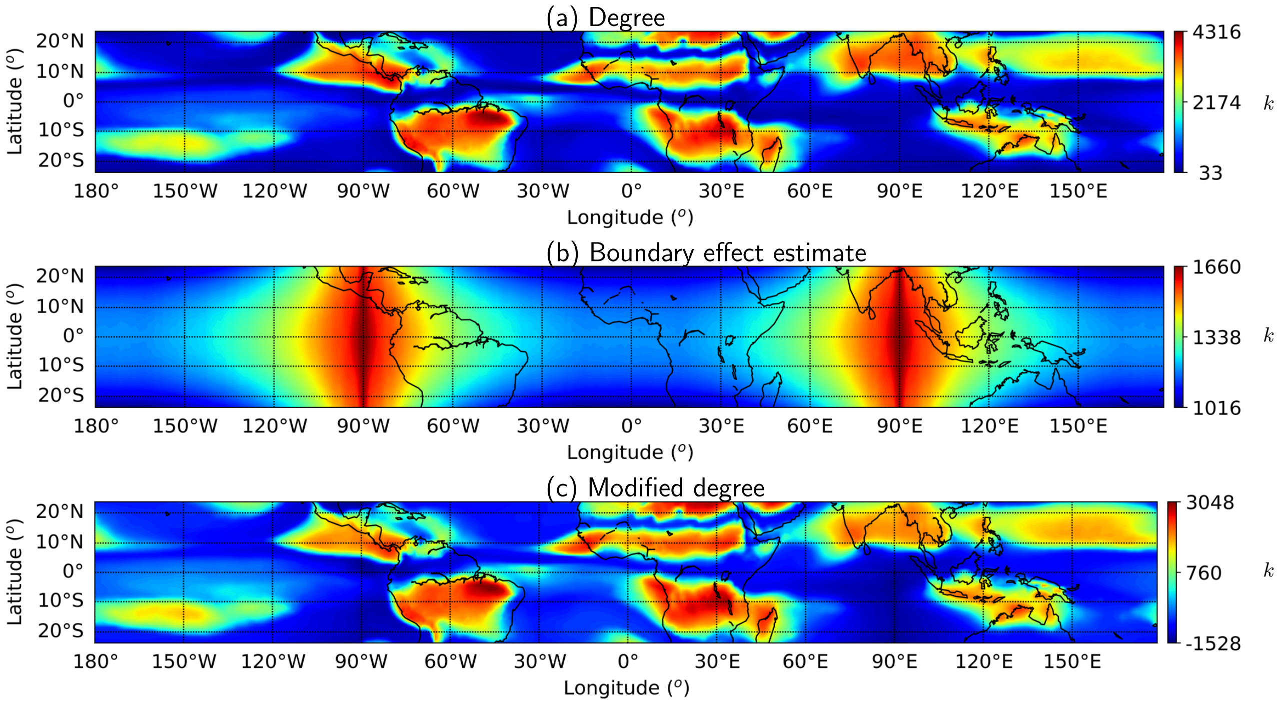

Figure 2: **Boundary effects on the spatial distribution of degree (k) due to spatial embedding. (a)** original degree, **(b)** boundary effect estimate from mean of 1000 surrogates and **(c)** modified degree obtained by subtracting the boundary effect estimate from the original degree.

The impact of Sahara desert region

The connections to the nodes belonging to the Sahara desert region have been removed while examining the intra-community connections of the Southern hemisphere (SH) community. Figure 3 shows the intra-community connections of the SH community with the nodes belonging to the Sahara region. We observe that the nodes in the Sahara region are connected to other regions in the SH community, such as southern Africa, South America, the Maritime continent, and the Pacific Ocean. As discussed in the paper, the $\widetilde{OLR}$ dynamics over these regions is associated with high convective activity and cloudiness. In contrast, the Sahara desert is devoid of such activities. Since the $\widetilde{OLR}$ dynamics over the Sahara desert and other regions are due to different physical mechanisms, we remove the connections of the nodes in the Sahara region while analysing intra-community connections.

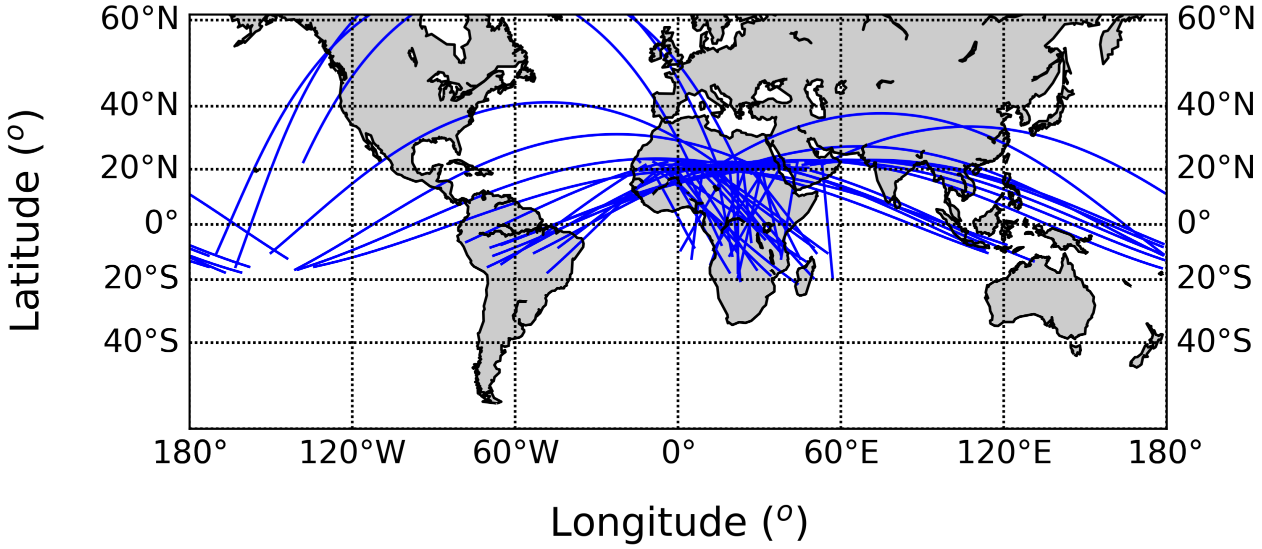

Figure 3: **Intra-community connections of the SH community:** connections with the Sahara desert region. The connections are shown using the greater circles. Please note that connections have been under-sampled for better visualization. The figure is created using the matplotlib basemap toolkit library (version 1.4.1, `matplotlib.org/basemap/stable/`) in Python.

Figure 4 shows the PDFs of intra-community degree (k^c) for the SH community with and without the nodes over the Sahara desert region. As expected, we observe a connectivity loss when connections with the Sahara desert region nodes are ignored. Loss in degree is observed at the higher end of the distribution. However, the rest of the distributions are in good agreement.

Testing for multiple comparison biases

It is important to correct for the biases arising due to multiple-testing/comparison in functional complex networks, where connections are established using statistical similarity measures. We use the method proposed by Boers et al. [2019] to correct multiple-comparison biases for spatially embedded functional climate networks. Their method considers that edges associated with physical climate mechanisms exhibit coherent spatial patterns, in contrast to edges associated with random coincidences. The approach entails constructing surrogate networks by randomly rewiring the links while preserving the link density of the original network. Next, we estimate each node's regional link density in the surrogate network. As suggested in Boers

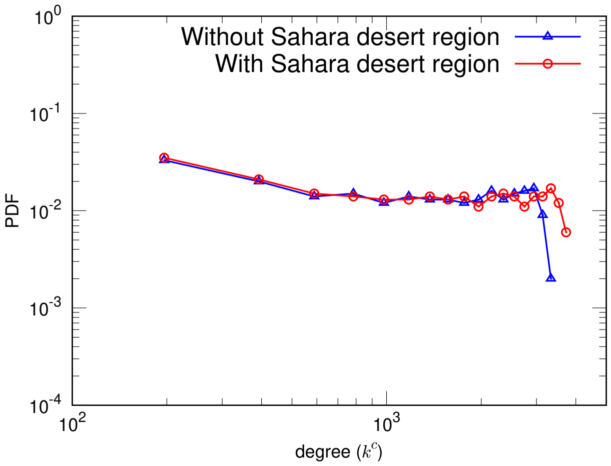

Figure 4: **Degree distribution:** pdf of intra-community degree for the SH community with and without nodes belonging to the Sahara desert region.

et al. [2019], we consider 1000 surrogates to obtain the null distribution. Nodes with a regional link density, estimated for the original network, greater than 99.9th percentile of the null-model distribution are considered significant.

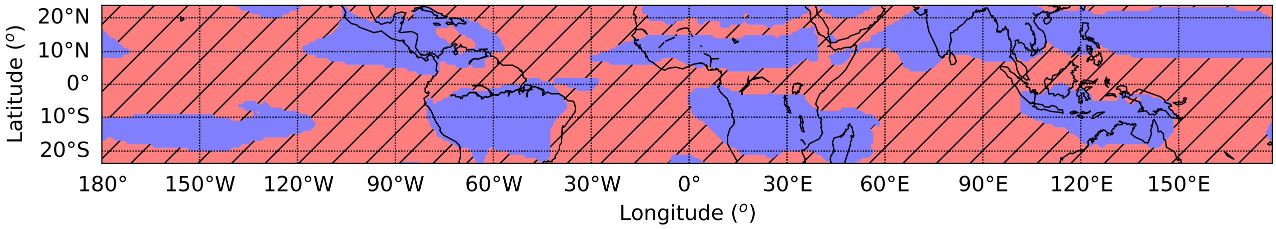

Figure 5: **Classification of nodes according to the multiple comparison testing approach by Boers et al. [2019]:** nodes in the blue region have a regional link density higher than the 99.9th percentile of the null distribution. In contrast, those in red-hatched regions have lower.

Figure 5 shows the significant (blue region) and insignificant (red hatched region) nodes. We observe from the figure that significant nodes correspond to the Northern (NH) and Southern Hemisphere (SH) communities (see Figure 2 of the manuscript). These communities have a high link density and also contain long-range teleconnections ($LD > 2500\ km$) [Boers et al., 2019] (see figure 3 of the manuscript). On the other hand, many insignificant nodes belong to communities with low link density and short-range links, such as the Equatorial Pacific and Atlantic Oceans (EPAO) and Equatorial Indian and Pacific Oceans (EIPO) communities (see Table 1 of the manuscript). Figure 6 compares the pdfs of link distance for the original network and the network consisting of just the significant nodes, referred to as the modified network. We can clearly observe the drop in the number of short-range links in the pdf of the modified network compared to that of the original network. Meanwhile, the distributions for long-range links ($LD > 2500\ km$) are in good agreement.

We observe that the insignificant nodes belong to communities with low intracommunity link density (ρ). Low ρ indicates that these communities are sparsely connected, which we attribute to incoherent spatio-temporal dynamics of $\widetilde{OLR}$ occurring in these regions. Regions encompassed by the EIPO and EPAO communities are prone to regional and mesoscale climate oscillations that enhance variability and incoherence. For example, the EIPO community experiences convective activities associated with boreal summer intraseasonal oscillations (BSISO)

and Madden Julian oscillations (MJO) [Kikuchi, 2021].

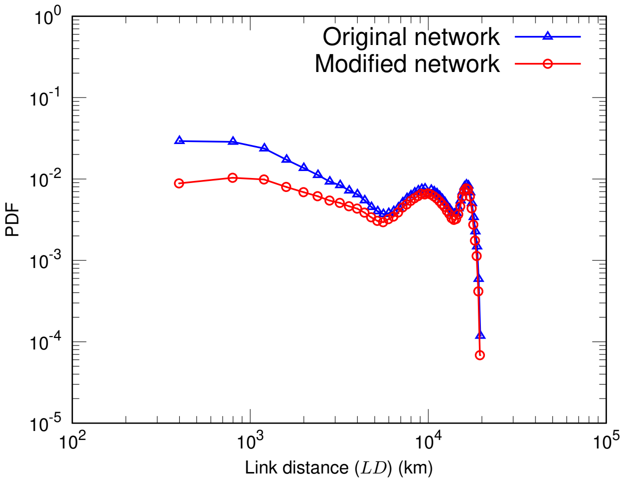

Figure 6: **Link distance distribution:** pdfs of link distance (LD) for the original and modified networks.

One of the primary objectives of this work is to classify tropical meteorology, which consists of rich phenomena across a wide range of spatio-temporal scales. Our classification unravels the regions of large-scale organized coherent OLR dynamics due to the planetry scale intertropical convergence zone (ITCZ) as well as the regions of incoherent dynamics due to mesoscale convective systems, intraseasonal oscillations and many others. As demonstrated above, multiple-comparison tests are very restrictive, and adopting them would lead to excluding nodes that do not exhibit coherent spatial patterns [Boers et al., 2019]. Identifying incoherent regions is also important. Activities occurring here are often responsible for extreme events such as excessive rainfall/droughts and climate variability in the tropics. For example, convective activity associated with BSISO originates in the Indian Ocean (EIPO community). It moves northward towards India, disturbing the rainfall pattern during the Indian monsoon. Therefore, we do not adopt multiple testing in this study.

Effect of spatial resolution

We examine the effect of finite sampling on the Salton similarity index (S) by performing the analysis on a spatial grid of higher resolution than the original one. We calculate the difference in the network topology between decade-2 ($2002-2011$) and decade-3 ($2012-2021$) networks. The finer spatial grid has spatial resolution of $0.5^\circ \times 0.5^\circ$ while the original grid is coarser with resolution of $1^\circ \times 1^\circ$. Figure 7 compares the S estimated on the finer and coarser spatial grids for decade-2 and decade-3 networks. Results from both spatial grids are in very good agreement. Therefore, we perform our analysis on the coarse spatial grids.

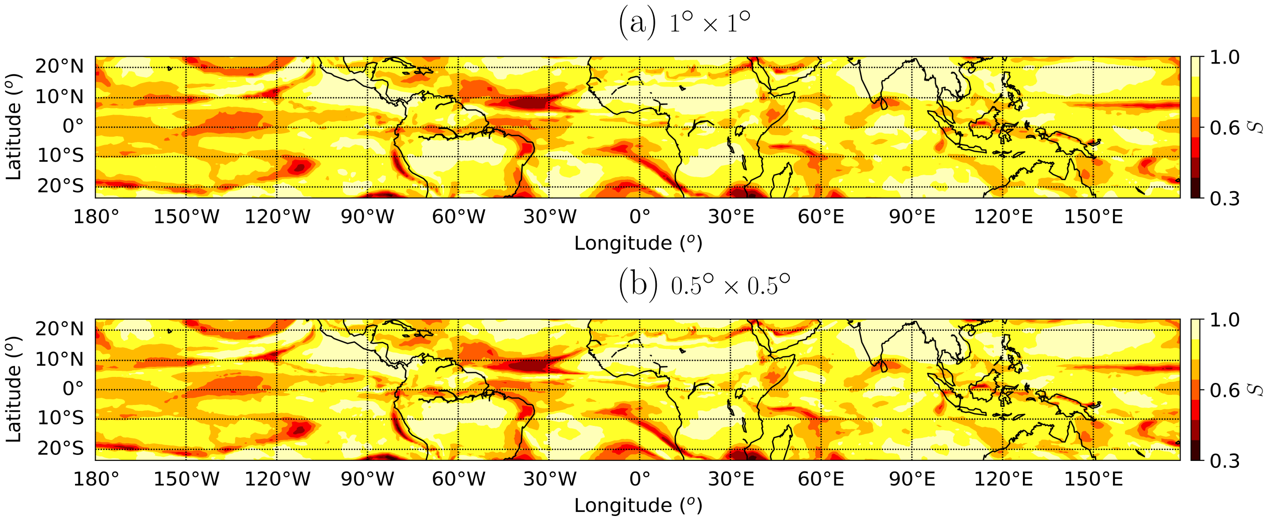

Figure 7: **Effect of spatial sampling on the Salton similarity index (S):** spatial distribution of S estimated for decade-2 to decade-3 $\widetilde{OLR}$ networks with spatial sampling resolution of (a) $1^\circ \times 1^\circ$ and (b) $0.5^\circ \times 0.5^\circ$.

References

S. Boccaletti, V. Latora, Y. Moreno, M. Chavez, and D. Hwang. Complex networks: Structure and dynamics. *Physics Reports*, 424(4-5):175–308, 2006.

N. Boers, B. Bookhagen, H. M. Barbosa, N. Marwan, J. Kurths, and J. Marengo. Prediction of extreme floods in the eastern Central Andes based on a complex networks approach. *Nature Communications*, 5(1):1–7, 2014.

N. Boers, B. Goswami, A. Rheinwalt, B. Bookhagen, B. Hoskins, and J. Kurths. Complex networks reveal global pattern of extreme-rainfall teleconnections. *Nature*, 566(7744):373–377, 2019.

J. F. Donges, Y. Zou, N. Marwan, and J. Kurths. Complex networks in climate dynamics. *The European Physical Journal Special Topics*, 174(1):157–179, 2009.

S. Gupta, N. Boers, F. Pappenberger, and J. Kurths. Complex network approach for detecting tropical cyclones. *Climate Dynamics*, 57:3355–3364, 2021.

M. D. Humphries and K. Gurney. Network 'small-world-ness': a quantitative method for determining canonical network equivalence. *PloS One*, 3(4):e0002051, 2008.

K. Kikuchi. The Boreal Summer Intraseasonal Oscillation (BSISO): A Review. *Journal of the Meteorological Society of Japan. Ser. II*, 99(4):933–972, 2021.

A. Rheinwalt, N. Marwan, J. Kurths, P. Werner, and F.-W. Gerstengarbe. Boundary effects in network measures of spatially embedded networks. *Europhysics Letters*, 100(2):28002, 2012.

A. A. Tsonis and P. J. Roebber. The architecture of the climate network. *Physica A: Statistical Mechanics and its Applications*, 333:497–504, 2004.

A. A. Tsonis and K. L. Swanson. Topology and predictability of El Niño and La Niña networks. *Physical Review Letters*, 100(22):228502, 2008.

E. van Diessen, W. J. Zweiphenning, F. E. Jansen, C. J. Stam, K. P. Braun, and W. M. Otte. Brain network organization in focal epilepsy: a systematic review and meta-analysis. *PloS One*, 9(12):e114606, 2014.

D. J. Watts and S. H. Strogatz. Collective dynamics of 'small-world' networks. *Nature*, 393 (6684):440–442, 1998.
